# Supplementary figures and images for: Inferring hidden structure in multilayered neural circuits
Source: PLoS Comput Biol. 2018 Aug 23;14(8):e1006291. doi: 10.1371/journal.pcbi.1006291 (PMC6124781; doi:10.1371/journal.pcbi.1006291)

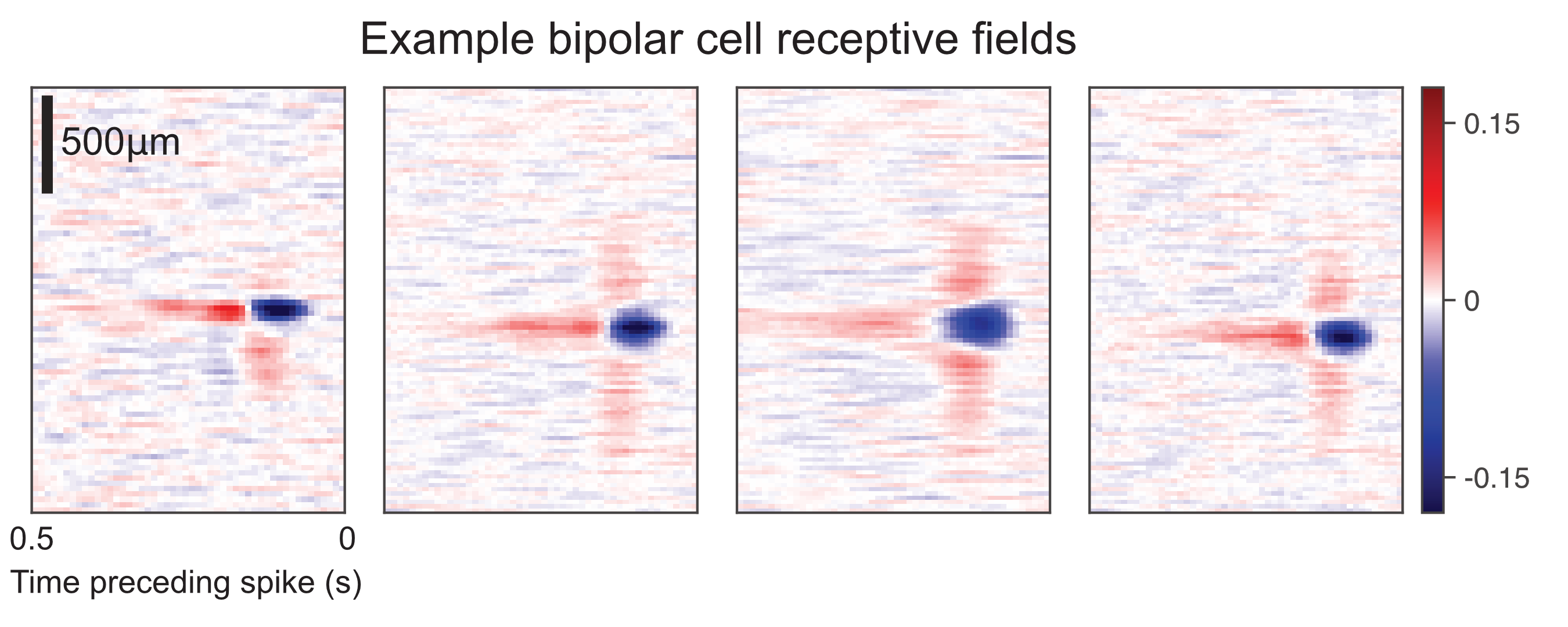

Supplement: S1 Fig — Each panel shows a spatiotemporal receptive field of an OFF bipolar cell recorded intracellularly from the salamander retina. (TIFF) [file pcbi.1006291.s001.tiff]

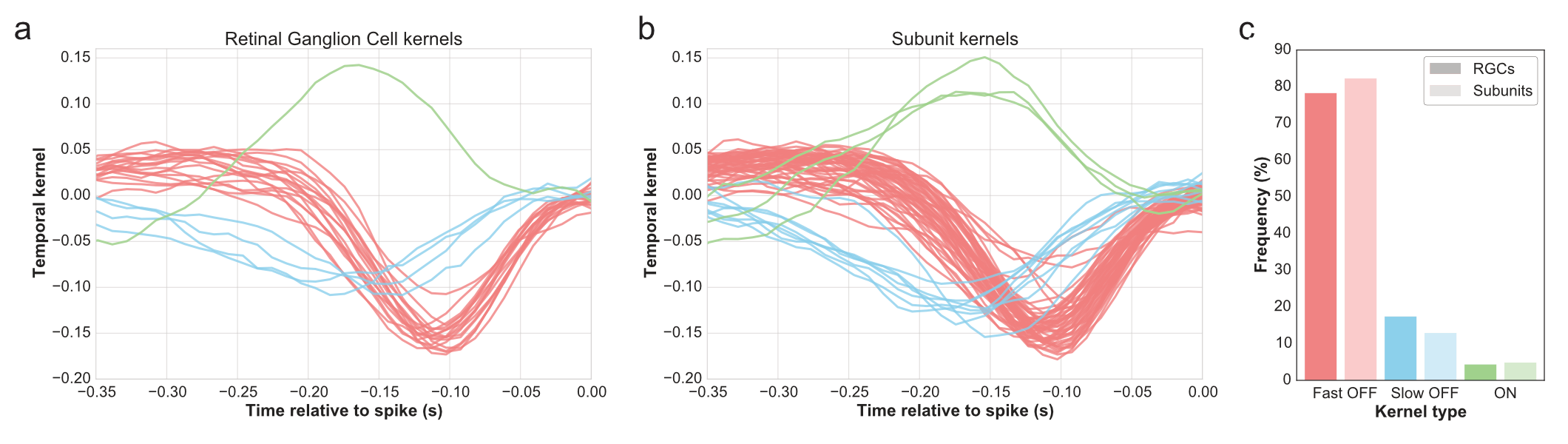

Supplement: S2 Fig — Cell type classification for salamander ganglion cell and subunit filters. (a) K-Means clustering applied to the temporal kernel (temporal component of the spatiotemporal receptive field) of n = 23 recorded retinal ganglion cells. (b) K-Means clustering applied to temporal kernels of n = 92 model-identified subunits. (c) Frequency of the different cell types, both for RGCs and subunits. (TIFF) [file pcbi.1006291.s002.tiff]
